# Patients’ Experiences of Digital Health Interventions for the Self-Management of Chronic Pain: Protocol for a Systematic Review and Thematic Synthesis

**DOI:** 10.2196/52469

**Published:** 2024-03-07

**Authors:** Ashleigh Main, Haruno McCartney, Maryam Ibrar, Harleen Kaur Rai, Fiona Muirhead, Alexandra Mavroeidi, Roma Maguire

**Affiliations:** 1 Department of Computer and Information Sciences University of Strathclyde Glasgow United Kingdom; 2 Physical Activity for Health, School of Psychological Sciences and Health University of Strathclyde Glasgow United Kingdom; 3 Department of Occupational Therapy and Human Nutrition and Dietetics, School of Health & Life Sciences Glasgow Caledonian University Glasgow United Kingdom

**Keywords:** chronic pain, digital health, digital tool, digital health intervention, mHealth, eHealth, pain-management, person-centered, experience, protocol, patients' experiences, patient experiences, self-management, systematic review, thematic synthesis, protocol.

## Abstract

**Background:**

Chronic pain is a highly prevalent condition that requires multidisciplinary treatment. However, in the United Kingdom, access to specialist pain clinics where patients can receive medical multidisciplinary treatment is limited, and provision varies between health boards. As such, self-management of chronic pain using digital tools has been gaining traction recently, but evidence of its effectiveness from clinical-based trials focuses mainly on quantitative outcomes.

**Objective:**

This systematic review aims to identify, appraise, and synthesize qualitative evidence on patients’ experiences with digital health interventions (DHIs) for the management of chronic pain.

**Methods:**

This systematic review will consider qualitative and mixed methods studies that explore the experience of patients (aged 18 years and older) with chronic pain engaging in DHIs to manage their pain. MEDLINE Ovid, PubMed, Embase, CINAHL, PsycINFO, and Scopus databases will be searched for published studies. The systematic review will be conducted in accordance with the ENTREQ (Enhancing Transparency in Reporting the Synthesis of Qualitative Research) guidelines. Following the 3-step thematic synthesis methodology of Thomas and Harden, titles and abstracts will be screened by 2 independent reviewers (AM and HM), and a third reviewer (MI or FM) will resolve any conflict that arises before the full-text screening. The Critical Appraisal Skills Programme checklist tool will be used to critically appraise the included studies. The extracted data will be imported to NVivo (QSR International), where thematic synthesis will be used to derive analytical themes from the included studies.

**Results:**

Themes that encapsulate the patient experience will be identified from qualitative evidence, and these themes will shed light on the perceived benefits and disadvantages, usability, acceptability, and the overall impact digital tools can have on the lives of those with chronic pain.

**Conclusions:**

This systematic review will identify, appraise, and synthesize the overall experience of patients engaging in DHI to manage a diverse range of chronic pain conditions. By elaborating the patient experience through qualitative analysis, the findings from this review will enhance our current understanding of the experiences of patients with chronic pain using digital tools for the self-management of their pain and highlight what person-centered elements are essential for future DHI development.

**Trial Registration:**

PROSPERO CRD42023445100; http://tinyurl.com/4z77khfs

**International Registered Report Identifier (IRRID):**

DERR1-10.2196/52469

## Introduction

### Background

Chronic pain is a major public health condition affecting more than 30% of the world’s population [1]. Defined as pain that persists beyond the expected recovery period of 12 weeks [2], chronic pain is a complex phenomenon, and the root causes are only partially understood [3]. Following recent developments, the *International Classification of Diseases* has adapted its definition of chronic pain to encompass both primary and secondary chronic pain. Chronic secondary pain is initially regarded as a symptom caused by an underlying condition or disease (such as cancer, rheumatoid arthritis, or endometriosis), whereas in chronic primary pain, pain emerges without any history of an injury or operation (eg, fibromyalgia, headache, and musculoskeletal pain) [2].

Chronic pain is a complex condition pertaining to biological, psychological, and social components [4]. A UK-based population study found that of 28 million adults affected by chronic pain, around 12% of people described their pain to be moderately or severely disabling [5]. In addition to the physical burden, psychological challenges are frequently associated with chronic pain with many individuals reporting a significantly deteriorated quality of life [6]. Chronic pain can also have a considerable socioeconomic impact, with pain impacting work effectiveness, which in many cases leads to unemployment [7].

To successfully manage the multifaceted nature of chronic pain, a multidisciplinary treatment approach is required. Substantial evidence supports that combining approaches across disciplines is the most effective treatment in addressing the biopsychosocial nature of chronic pain [8-12], although access to evidence-based multidisciplinary treatment is not always readily available [13]. Many patients with chronic pain rely on their general practitioner for advice [14], and those who do get referred to specialist pain clinics can wait months to years for assessment and treatment [13].

A means of overcoming the barriers of accessibility to multidisciplinary health care could be the delivery of self-management strategies using digital tools, which have become more prevalent than ever since the COVID-19 pandemic. Digitization has transformed the health care landscape by offering more accessible alternatives to conventional medical treatment [15]. Digital health interventions (DHIs) use a range of digital tools, such as smartphone apps, websites, and social media campaigns, to deliver self-management programs remotely [16]. Self-management strategies for chronic pain management include symptom tracking, physical activity, education, relaxation techniques, and cognitive behavioral therapy [17-20]. According to the Office of National Statistics, approximately 90% of private UK households have computers and mobile phones in 2022; therefore, digital health is accessible to almost everyone [21].

Research regarding the impact of digital tools has grown exponentially in recent years. A number of systematic reviews have evaluated the effectiveness of digital tools in areas such as chronic pain in general [16,22], specific chronic pain conditions [23,24], the impact of individual modalities such as smartphones [25,26], and specific age groups [27]. It is widely considered that such tools are effective in improving clinical outcomes such as pain intensity, pain interference, and improvements in quality of life [28].

Although there is substantial evidence on the positive effect digital tools can have on chronic pain patients’ symptoms, most research focuses on quantitative outcomes. Quantitative research is essential in supporting evidence-based practice, which emphasizes findings from well-designed research to provide high-quality patient care [29]. However, it has been argued that quantitative evidence from clinical trials does not take into account patients’ individual experiences, thus overlooking the complex nature of chronic pain [30]. In response to standard evidence-based practice, there has been a shift to person-centered care, which has transformed the health care system. Person-centered care was first described as “understanding the patient as a unique human being” [31], where the sole focus is on the individual, and treatment can be tailored to a patient’s needs. The World Health Organization (WHO) guidelines advise person-centered care as a core element of good quality health care [2], and studies have shown that person-centered care significantly improves clinical outcomes [32].

A systematic review of qualitative studies by Fernandes et al [33] evaluated enablers and barriers to telehealth interventions for individuals with musculoskeletal pain, and Svendsen et al [34] explored engagement strategies, facilitators, and barriers to the use of DHIs for low back pain management. However, both qualitative reviews focus on barriers and facilitators to DHI engagement rather than overall patient experience, and both studies target a specific condition as opposed to a diverse range of chronic pain conditions. Despite the abundance of qualitative literature exploring the experiences of patients with chronic pain with DHIs, the evidence that encapsulates the experience as a whole has yet to be synthesized.

To address this gap in the research, this review will identify, appraise, and synthesize qualitative data, through a thematic synthesis approach, to provide a detailed account of how patients with chronic pain experience DHIs, and if and why they experience changes and improvements in patient-reported outcomes. Thematic synthesis was developed to address questions concerning intervention need, appropriateness, and acceptability and can produce results that have the potential to inform the design and practice of interventions [35]. Descriptive explanations of patients with chronic pain views could uncover a better understanding of DHI lived experiences from the point of view of the end user—for whom the treatment is designed. Therefore, the findings from this review could have the potential to enhance our understanding of how DHIs impact patients with chronic pain and emphasize what patient-centered aspects are important for future DHI development.

### Objectives

This review aims to identify, appraise, and synthesize existing qualitative evidence on patients’ experiences with a DHI for the self-management of chronic pain.

## Methods

### Overview

The proposed review will be conducted in accordance with “ENTREQ (Enhancing Transparency in Reporting the Synthesis of Qualitative Research) guidelines [36].

### Search Strategy

The search strategy and eligibility criteria will be based on the SPIDER (Sample, Phenomenon of Interest, Design, Evaluation and Research) framework [37]. This framework was deemed the most suitable for answering the research question as it has been adapted for clarity in nonquantitative research by outlining key characteristics of qualitative research questions [37].

Keywords used in the search strategy adhere to each of the key characteristics of the SPIDER framework. These include chronic pain as the sample (“fibromyalgia” and “rheumatoid arthritis”), DHIs as the phenomenon of interest (“mHealth” and “telemedicine”), qualitative methods as the design and research type (“qualitative” and “thematic analysis”), and patient experience as the evaluated outcomes (“patient satisfaction” and “patient attitudes”).

The search strategy will involve an initial search of electronic databases, followed by the analysis of keywords in the titles and abstracts of each database search. The search will then continue to the reference lists of selected articles to identify additional studies not located through the electronic database search. The strategy will aim to locate published articles—gray literature and unpublished articles will be excluded from the review. Due to the rapidly evolving nature of digital technology, the search will be limited to articles published within the last 10 years in order to capture the most relevant literature (2013-2023). The search will also be limited to articles published in the English language. The search strategy will be adapted for each electronic database. A detailed search strategy for each database is presented in Multimedia Appendix 1.

### Information Sources

A literature search for qualitative and mixed methods studies will be conducted on the following electronic databases: Embase, MEDLINE, PubMed, CINAHL, PsycINFO, and Scopus. These databases have been chosen as they encompass nursing, medicine, social sciences, and psychology literature, which are deemed the most appropriate for answering the research question concerning patient experience and chronic pain.

### Eligibility Criteria

Inclusion criteria will follow the SPIDER [37] framework. Textbox 1 shows the list of inclusion and exclusion criteria.

**Inclusion and exclusion criteria based on the SPIDER (Sample, Phenomenon of Interest, Design, Evaluation and Research) framework.**

**Inclusion criteria**

- Papers reporting on participants (aged ≥18 years old) with a diagnosis of chronic pain
- Papers that report on digital health interventions to deliver self-management strategies for chronic pain
- Qualitative studies and mixed methods studies with a qualitative component
- Papers that report on patient experience of participating in a digital health intervention through qualitative data
- Papers published in the English language

**Exclusion criteria**

- Papers reporting on participants <18 years old
- Papers that have not specified that the participants have a chronic pain diagnosis
- Papers that include participants with chronic pain that does not exceed 12 weeks
- Papers that report on digital health interventions that incorporate external involvement
- Quantitative studies, quantitative components from mixed methods studies, gray literature, protocols, dissertations, and other reviews
- Papers that report data from participants who did not actively participate in the intervention
- Papers published in a language other than English

### Sample

The review will include studies that involve patients older than age of 18 years with a diagnosis of chronic pain, who have participated in a DHI that delivered self-management strategies. Studies exploring the experience of children, adolescents, clinicians, or health professionals will be excluded. The definition of chronic pain will be aligned with the revised *International Classification of Diseases, 11th Revision* classification: pain that persists or recurs for more than 3 months [2], including both primary and secondary chronic pain. The included conditions are based on the National Institute for Health and Care Excellence guidelines for assessing and managing both primary and secondary chronic pain [3].

### Phenomenon of Interest

The phenomenon of interest explored in this review will be DHIs that deliver self-management strategies for chronic pain. Digital interventions regardless of platform (eg, telephone, web-based smartphone app, and social media) or self-management strategy (eg, exercise, education, relaxation, and meditation) will be included. Due to the terminological inconsistency existent in the current literature, the review will not distinguish among telehealth, telemedicine, eHealth, mobile health, or other similar terms. This review will use the term “digital health” to encompass all related terms.

### Design and Research Type

The review will consider studies that explore patients’ experiences of participating in a DHI. Qualitative data regarding patient experience collected through surveys, questionnaires, focus groups, group and individual interviews, and observational data will be examined, and this may have been collected remotely or face-to-face or in a group or one-to-one setting. Mixed methods studies with a qualitative component will be included; the quantitative data from these studies will not be used in this review. Protocols, dissertations, gray literature, and other reviews will be excluded.

### Evaluated Outcomes

The outcome evaluated in this review will be patients’ experiences of participating in DHIs. This will be in the form of qualitative data collected via questionnaires, focus groups, surveys, observational data, and interviews. Patients must have participated in the intervention either entirely or partially, for example, patients who started a digital intervention and followed through until completion, and patients who started a digital intervention and did not complete it in its entirety. Studies exploring patients’ opinions or perceptions of what an intervention should involve without active participation will be excluded.

### Data Extraction

Articles identified from the database search will be uploaded to EndNote (version 20; Clarivate), and any duplicates will be removed. Two independent reviewers (AM and HM) will then screen the titles and abstracts in Rayyan for papers that meet the inclusion criteria. The data extracted will adhere to the SPIDER tool, specifically outlining the sample, phenomenon of interest, and outcomes of significance to the objective of this review [37]. Any uncertainty between reviewers will be resolved by discussion, and if necessary, a third reviewer (MI or FM) will be requested to resolve any disagreements that persist. This process will be repeated for the papers included for full-text screening. Subsequently, the remaining papers will be included in the review. Each paper will be uploaded to NVivo (version 12) as this will facilitate the researcher’s ability to perform data synthesis systematically and rigorously. The full data extraction table will include the author, year of publication, country design, data collection method, participant characteristics, type and description of the intervention, and potential themes.

### Strategy for Data Synthesis

The results and discussion section of each included paper will be imported verbatim to NVivo, where a 3-step thematic synthesis approach based on the methodology described by Thomas and Harden will be used [38].

1. The first step will involve line-by-line coding of the results and discussion sections to identify contextual information on patient experiences with DHIs for the self-management of chronic pain.
2. Subsequently, similarly coded data will be clustered together to generate “descriptive themes.” Descriptive themes will be based on verbatim data from the selected studies. Reviewers will reassess these codes to ensure the data are captured accurately.
3. Consequently, using inductive reasoning to make inferences from previous codes, reviewers will identify “analytical themes” about the experiences captured in the descriptive themes. These themes will be relevant to the key aim of this meta-synthesis. These themes are comprised based on inferences made from the data and, therefore, will be conducted independently by 1 reviewer (AM).

### Risk of Bias or Critical Appraisal for Included Studies

The Critical Appraisal Skills Programme (CASP) checklist tool will be used to assess the methodological quality of the included studies. The CASP tool was deemed appropriate for the context of this review as it is endorsed by Cochrane and the WHO [39] and is the most frequently used tool for quality appraisal in health-related qualitative evidence syntheses [40]. Two reviewers (AM and HM) will assess the quality of the included papers independently, and any discrepancies that arise between reviewers will be resolved through discussion. If an agreement is not met, an additional reviewer (MI or FM) will be consulted until a consensus has been reached.

### Ethical Considerations

Ethical approval is not required for conducting the systematic review as the research does not directly involve human participants or access to personal or identifiable data. The findings from this review will be disseminated to a broad range of stakeholders, including academics, clinicians, and policy makers. The findings will also be published in accredited peer-reviewed journals. This protocol is part of a larger PhD project; therefore, the findings will also be included as part of the thesis.

## Results

This review will synthesize qualitative literature on patients’ experiences of participating in a DHI for the self-management of chronic pain. Using thematic synthesis, an adapted version of Braun and Clark’s thematic analysis developed for the purpose of secondary data synthesis [38,41], analytical themes will be derived from the included studies. We anticipate that the results from this review will characterize the patient experience of DHIs for the self-management of chronic pain and explain the impact digital tools have on the lives of those with chronic pain. These findings will emphasize what patient-centered aspects are essential for future DHI development, such as how they engaged with DHIs, perceived advantages and disadvantages, which aspects of DHIs they felt worked well and which could be improved, and insights on acceptability and usability. Evidence-based practice emphasizes evidence from well-designed research. However, it has been argued that this approach does not value individual experience and subsequently does not effectively represent the complexity of the chronic pain experience [38]. The results of this qualitative systematic review will enhance our understanding of the way patients experience DHIs for the self-management of chronic pain by emphasizing individual experience from a person-centered approach.

## Discussion

### Principal Findings

Previous systematic reviews have demonstrated the effectiveness of DHIs in improving patients’ outcomes across various health conditions, including chronic pain in general [16,22] as well as specific chronic pain conditions like musculoskeletal pain [23]. However, there is a lack of research evaluating qualitative studies that explore how patients with chronic pain experience such improvements in DHIs. A qualitative exploration of experiences has the potential to generate an in-depth, representative, conceptual understanding of how patients exhibit such improvements in outcomes. Themes that characterize the patient experience of participating in DHIs for the self-management of chronic pain will be identified to capsulate the impact DHIs have on individuals living with chronic pain.

### Strengths and Limitations

The comprehensive systematic approach to the current review is a major strength of this research. The search strategy and inclusion criteria are based on SPIDER, a framework adapted for clarity in qualitative research by defining crucial characteristics of qualitative research questions [37]. The search strategies for each database were developed with the help of 2 specialist librarians (see Multimedia Appendix 1), and search terms regarding chronic pain were taken from the National Institute for Health and Care Excellence guidelines for the management of chronic pain [3]. The review will be guided by the ENTREQ checklist to ensure a rigorous systematic approach is taken to synthesize the qualitative evidence [36]. The quality of included studies will be assessed using the CASP checklist tool, which has been endorsed by Cochrane and the WHO [39]. Following the 3-step thematic synthesis approach based on the methodology described by Thomas and Harden, extracted data will be reviewed by 2 independent researchers (AM and HM), and a third reviewer (MI or FM) will be consulted to resolve any issues that arise.

Limitations of this review include the exclusion of studies published in a language other than English, as well as gray literature, both of which could have valuable contributions to the research outcomes. Furthermore, although thematic synthesis is a powerful method that can draw overall conclusions on a specific topic, the raw data from each included study are not analyzed, which could undermine the richness of the data from each primary study.

### Conclusions

This systematic review will be the first to synthesize the overall experience of patients engaging in DHIs to manage a diverse range of chronic pain conditions. The in-depth analysis provided by qualitative data will enhance our current knowledge, representing the point of view of the end user—for whom the digital interventions are designed to treat. By elaborating the patient experience through qualitative analysis, the findings from this review have the potential to inform the future development of DHIs by highlighting which person-centered aspects are crucial to effectively manage symptoms of chronic pain.

## Appendix

Multimedia Appendix 1 Search strategy for each database.

## Abbreviations

CASP: Critical Appraisal Skills Programme
DHI: digital health intervention
ENTREQ: Enhancing Transparency in Reporting the Synthesis of Qualitative Research
SPIDER: Sample, Phenomenon of Interest, Design, Evaluation and Research
WHO: World Health Organization

## Data Availability

Data sharing is not applicable to the current article as no data were generated or analyzed during this study.
