# Supplementary material for: Patients’ Experiences of Digital Health Interventions for the Self-Management of Chronic Pain: Protocol for a Systematic Review and Thematic Synthesis
Source: JMIR Res Protoc. 2024 Mar 7;13:e52469. doi: 10.2196/52469 (PMC10958340; doi:10.2196/52469)
Supplement: Multimedia Appendix 1 [file resprot_v13i1e52469_app1.docx]

**EMBASE (Ovid)**

1. chronic pain/
2. (chronic adj5 pain).mp.
3. (chronic pain or persistent pain or long-term pain or fibromyalgia or rheumatoid arthritis or neuro* pain or musculoskeletal pain* or orofacial pain or visceral pain or endometriosis or headache or irritable bowel syndrome or back pain* or low back pain or neck pain or joint pain* sciatica or osteoarthritis or cancer pain or post surg* pain or post trauma* pain).mp.
4. Telemedicine/
5. telehealth/
6. (health adj5 technolog*).mp.
7. (health adj5 app*).mp.
8. (mhealth or ehealth or telenursing or telerehabilitation or digital health or digital intervention or remote consultation or electronic health or internet health).mp.
9. patient satisfaction/
10. patient attitude/
11. (experience* or perspective* or perception* or accept* or satisf* or view* or attitude*).mp.
12. qualitative research/
13. (qualitative or grounded theory or ethnography or thematic analysis or mixed method* or focus group* or interview*).mp.
14. or/1-3
15. or/4-8
16. or/9-11
17. or/12-13
18. and/14-17
19. limit 18 to (english and last 10 years)

**Medline (Ovid)**

1. chronic pain/
2. (chronic adj5 pain).mp.
3. (chronic pain or persistent pain or long-term pain or fibromyalgia or rheumatoid arthritis or neuro* pain or musculoskeletal pain* or orofacial pain or visceral pain or endometriosis or headache or irritable bowel syndrome or back pain* or low back pain or neck pain or joint pain* sciatica or osteoarthritis or cancer pain or post surg* pain or post trauma* pain).mp.
4. Telemedicine/
5. (health adj5 technolog*).mp.
6. (health adj5 app*).mp.
7. (mhealth or ehealth or telenursing or telerehabilitation or digital health or digital intervention or remote consultation or electronic health or internet health).mp.
8. Patient Satisfaction/
9. Attitude to Health/
10. (experience* or perspective* or perception* or accept* or satisf* or view* or attitude*).mp.
11. qualitative research/
12. (qualitative or grounded theory or ethnography or thematic analysis or mixed method* or focus group* or interview*).mp.
13. or/1-3
14. or/4-7
15. or/8-10
16. or/11-12
17. and/13-16
18. limit 17 to (english and last 10 years)

**CINAHL (EBSCOhost)**

- MH "chronic pain" OR TI ( "chronic pain" or "persistent pain" or "long-term pain" or fibromyalgia or "rheumatoid arthritis" or osteoarthritis or "neuro* pain" or "musculoskeletal pain*" or "orofacial pain" or "visceral pain" or endometriosis or headache or "irritable bowel syndrome" or "back pain*" or "low* back pain" or "neck pain" or "joint pain*" or sciatica or "cancer pain" or "post surg* pain" or "post trauma* pain" or "complex regional pain" or "chronic primary headache" or "chronic primary visceral pain" or "chronic musculoskeletal pain" ) OR AB ( "chronic pain" or "persistent pain" or "long-term pain" or fibromyalgia or "rheumatoid arthritis" or osteoarthritis or "neuro* pain" or "musculoskeletal pain*" or "orofacial pain" or "visceral pain" or endometriosis or headache or "irritable bowel syndrome" or "back pain*" or "low* back pain" or "neck pain" or "joint pain*" or sciatica or "cancer pain" or "post surg* pain" or "post trauma* pain" or "complex regional pain" or "chronic primary headache" or "chronic primary visceral pain" or "chronic musculoskeletal pain" ) OR SU ( "chronic pain" or "persistent pain" or "long-term pain" or fibromyalgia or "rheumatoid arthritis" or osteoarthritis or "neuro* pain" or "musculoskeletal pain*" or "orofacial pain" or "visceral pain" or endometriosis or headache or "irritable bowel syndrome" or "back pain*" or "low* back pain" or "neck pain" or "joint pain*" or sciatica or "cancer pain" or "post surg* pain" or "post trauma* pain" or "complex regional pain" or "chronic primary headache" or "chronic primary visceral pain" or "chronic musculoskeletal pain" )

AND

MH ( "telehealth" or "digital health" ) OR TI ( mhealth or ehealth or telenursing or telerehabilitation or "digital intervention" or "remote consultation" or "electronic health" or "internet health" or "digital health" or “health technolog*" OR "health management app*" OR "digital health programme" OR "digital health app*" ) OR AB ( mhealth or ehealth or telenursing or telerehabilitation or "digital intervention" or "remote consultation" or "electronic health" or "internet health" or "digital health" or “health technolog*" OR "health management app*" OR "digital health programme" OR "digital health app*" ) OR SU ( mhealth or ehealth or telenursing or telerehabilitation or "digital intervention" or "remote consultation" or "electronic health" or "internet health" or "digital health" or “health technolog*" OR "health management app*" OR "digital health programme" OR "digital health app*" )

AND

MH ( "patient satisfaction" or "patient attitudes" ) OR TI ( experience* or perspective* or perception* or accept* or satisf* or view* or attitude* ) OR AB ( experience* or perspective* or perception* or accept* or satisf* or view* or attitude* ) OR SU ( experience* or perspective* or perception* or accept* or satisf* or view* or attitude* )

AND

MH qualitative research OR TI ( qualitative or "grounded theory" or ethnography or "thematic analysis" or "mixed method*" or "focus group*" or interview* ) OR AB ( qualitative or "grounded theory" or ethnography or "thematic analysis" or "mixed method*" or "focus group*" or interview* ) OR SU ( qualitative or "grounded theory" or ethnography or "thematic analysis" or "mixed method*" or "focus group*" or interview* )

Limit to last 10 years and English language

**PsychInfo (EBSCOhost)**

MH "chronic pain" OR TI ( "chronic pain" or "persistent pain" or "long-term pain" or fibromyalgia or "rheumatoid arthritis" or osteoarthritis or "neuro* pain" or "musculoskeletal pain*" or "orofacial pain" or "visceral pain" or endometriosis or headache or "irritable bowel syndrome" or "back pain*" or "low* back pain" or "neck pain" or "joint pain*" or sciatica or "cancer pain" or "post surg* pain" or "post trauma* pain" or "complex regional pain" or "chronic primary headache" or "chronic primary visceral pain" or "chronic musculoskeletal pain" ) OR AB ( "chronic pain" or "persistent pain" or "long-term pain" or fibromyalgia or "rheumatoid arthritis" or osteoarthritis or "neuro* pain" or "musculoskeletal pain*" or "orofacial pain" or "visceral pain" or endometriosis or headache or "irritable bowel syndrome" or "back pain*" or "low* back pain" or "neck pain" or "joint pain*" or sciatica or "cancer pain" or "post surg* pain" or "post trauma* pain" or "complex regional pain" or "chronic primary headache" or "chronic primary visceral pain" or "chronic musculoskeletal pain" ) OR SU ( "chronic pain" or "persistent pain" or "long-term pain" or fibromyalgia or "rheumatoid arthritis" or osteoarthritis or "neuro* pain" or "musculoskeletal pain*" or "orofacial pain" or "visceral pain" or endometriosis or headache or "irritable bowel syndrome" or "back pain*" or "low* back pain" or "neck pain" or "joint pain*" or sciatica or "cancer pain" or "post surg* pain" or "post trauma* pain" or "complex regional pain" or "chronic primary headache" or "chronic primary visceral pain" or "chronic musculoskeletal pain" )

AND

MH ( "telemedicine" or "mobile health" ) OR TI ( mhealth or ehealth or telenursing or telerehabilitation or "digital intervention" or "remote consultation" or "electronic health" or "internet health" or "digital health" or “health technolog*" OR "health management app*" OR "digital health programme" OR "digital health app*" ) OR AB ( mhealth or ehealth or telenursing or telerehabilitation or "digital intervention" or "remote consultation" or "electronic health" or "internet health" or "digital health" or “health technolog*" OR "health management app*" OR "digital health programme" OR "digital health app*" ) OR SU ( mhealth or ehealth or telenursing or telerehabilitation or "digital intervention" or "remote consultation" or "electronic health" or "internet health" or "digital health" or “health technolog*" OR "health management app*" OR "digital health programme" OR "digital health app*" )

AND

MH ( "patient satisfaction" ) OR TI ( experience* or perspective* or perception* or accept* or satisf* or view* or attitude* ) OR AB ( experience* or perspective* or perception* or accept* or satisf* or view* or attitude* ) OR SU ( experience* or perspective* or perception* or accept* or satisf* or view* or attitude* )

AND

MH qualitative research OR TI ( qualitative or "grounded theory" or ethnography or "thematic analysis" or "mixed method*" or "focus group*" or interview* ) OR AB ( qualitative or "grounded theory" or ethnography or "thematic analysis" or "mixed method*" or "focus group*" or interview* ) OR SU ( qualitative or "grounded theory" or ethnography or "thematic analysis" or "mixed method*" or "focus group*" or interview* )

Limit to last 10 years and English language

**SCOPUS**

( TITLE-ABS-KEY ( "chronic pain" OR "persistent pain" OR "long-term pain" OR fibromyalgia OR "rheumatoid arthritis" OR osteoarthritis OR "neuro* pain" OR "musculoskeletal pain*" OR "orofacial pain" OR "visceral pain" OR endometriosis OR headache OR "irritable bowel syndrome" OR "back pain*" OR "low* back pain" OR "neck pain" OR "joint pain*" OR sciatica OR "cancer pain" OR "post surg* pain" OR "post trauma* pain" OR "complex regional pain" OR "chronic primary headache" OR "chronic primary visceral pain" OR "chronic musculoskeletal pain" )

AND

TITLE-ABS-KEY ( experience* OR perspective* OR perception* OR accept* OR satisf* OR view* OR attitude* )

AND

TITLE-ABS-KEY ( qualitative OR "grounded theory" OR ethnography OR "thematic analysis" OR "mixed method*" OR "focus group*" OR interview* )

AND

TITLE-ABS-KEY ( mhealth OR ehealth OR telenursing OR telerehabilitation OR "digital intervention" OR "remote consultation" OR "electronic health" OR "internet health" OR "digital health" OR "health technolog*" OR "health management app*" OR "digital health program*" ) )

AND

PUBYEAR > 2013 AND PUBYEAR < 2023 AND ( LIMIT-TO ( LANGUAGE , "english" ) )

**Pubmed**

("chronic pain"[MeSH Terms] OR "chronic pain"[Title/Abstract] OR "persistent pain"[Title/Abstract] OR "long-term pain"[Title/Abstract] OR "fibromyalgia"[Title/Abstract] OR "rheumatoid arthritis"[Title/Abstract] OR "osteoarthritis"[Title/Abstract] OR "neuro pain"[Title/Abstract] OR "musculoskeletal pain*"[Title/Abstract] OR "orofacial pain"[Title/Abstract] OR "visceral pain"[Title/Abstract] OR "endometriosis"[Title/Abstract] OR "headache"[Title/Abstract] OR "irritable bowel syndrome"[Title/Abstract] OR "back pain*"[Title/Abstract] OR "low back pain"[Title/Abstract] OR "neck pain"[Title/Abstract] OR "joint pain*"[Title/Abstract] OR "sciatica"[Title/Abstract] OR "cancer pain"[Title/Abstract] OR "post trauma pain"[Title/Abstract] OR "complex regional pain"[Title/Abstract] OR "chronic primary headache"[Title/Abstract] OR "chronic primary visceral pain"[Title/Abstract] OR "chronic musculoskeletal pain"[Title/Abstract])

AND

(“telemedicine”[MeSH Terms] OR “telemedicine”[MeSH Terms] OR “digital health”[MeSH Terms] OR “mhealth”[Title/Abstract] OR “ehealth”[Title/Abstract] OR “telenursing”[Title/Abstract] OR “telerehabilitation”[Title/Abstract] OR “digital intervention”[Title/Abstract] OR “remote consultation”[Title/Abstract] OR “electronic health”[Title/Abstract] OR “internet health”[Title/Abstract] OR “digital health”[Title/Abstract] OR “health technolog*”[Title/Abstract] OR “health management app*”[Title/Abstract] OR “digital health program*”[Title/Abstract])

AND

("experience*"[Title/Abstract] OR "perspective*"[Title/Abstract] OR "perception*"[Title/Abstract] OR "accept*"[Title/Abstract] OR "satisf*"[Title/Abstract] OR "view*"[Title/Abstract] OR "attitude*"[Title/Abstract])

AND

("qualitative"[Title/Abstract] OR "grounded theory"[Title/Abstract] OR "ethnography"[Title/Abstract] OR "thematic analysis"[Title/Abstract] OR "mixed method*"[Title/Abstract] OR "focus group*"[Title/Abstract] OR "interview*"[Title/Abstract])

AND

("2014/01/26 00:00":"3000/01/01 05:00"[Date - Publication] AND "english"[Language])) AND ((y_10[Filter]) AND (english[Filter]))
